# Prognostic markers for survival in patients with oligodendroglial tumors; a single-institution review of 214 cases

**DOI:** 10.1371/journal.pone.0188419

**Published:** 2017-11-29

**Authors:** Maria Zetterling, Luwam Berhane, Irina Alafuzoff, Asgeir S. Jakola, Anja Smits

**Affiliations:** 1 Department of Neuroscience, Neurosurgery, Uppsala University, University Hospital, Uppsala, Sweden; 2 Department of Neuroscience, Neurology, Uppsala University, University Hospital, Uppsala, Sweden; 3 Department of Immunology, Genetics and Pathology, Experimental and clinical Oncology, Uppsala University, University Hospital, Uppsala, Sweden; 4 Institute of Neuroscience and Physiology, Department of Clinical Neuroscience, Sahlgrenska Academy, University of Gothenburg, Gothenburg, Sweden; 5 Department of Neurosurgery, Sahlgrenska University Hospital, Gothenburg, Sweden; 6 Department of Neurosurgery, St.Olavs Hospital, Trondheim, Norway; University of South Alabama Mitchell Cancer Institute, UNITED STATES

## Abstract

**Background:**

In the 2016 WHO classification, the diagnosis of oligodendroglioma has been restricted to IDH mutated, 1p19q codeleted tumors (IDHmut-codel). IDHmut oligoastrocytoma is now classified either as oligodendroglioma or astrocytoma based on presence of 1p19q codeletion. There is growing evidence that this molecular classification more closely reflects patient outcome. Due to the strong association between IDHmut-codel with oligodendroglial morphology, the additional impact of these markers on prognostic accuracy is largely unknown. Our aim was to assess the prognostic impact of IDHmut-codel in an unselected cohort of morphologically classified oligodendroglial tumors.

**Methods:**

We performed a retrospective chart review of oligodendroglial tumors (WHO grade II and III) operated since 1983. A total of 214 tumors were included, and molecular information was available for 96 tumors. The prognostic impact of IDHmut-codel together with clinical parameters was analyzed by multivariate Cox regression.

**Results:**

IDHmut-codel was registered in 64 tumors while for 150 tumors the molecular profile was negative for IDHmut-codel, unknown or incomplete. Comparison between the two groups showed that patients with IDHmut-codel tumors were younger (42 vs. 48 years), had more frequent frontal tumor location (48 vs. 33%) and presented more often with seizures (72 vs. 51%) and no signs of neurological impairment (14 vs. 30%) than patients harboring tumors with unknown or incomplete molecular profile. Multivariate survival analysis identified young age (HR 1.78 ≥ 40 years), the absence of neurological deficits or personality changes (HR 0.57), frontal tumor location (HR 0.64) and the presence of IDHmut-codel (HR 0.50) as independent predictors for longer survival, whereas tumor grade was not.

**Conclusion:**

In this unselected single-institution cohort, the presence of IDHmut-codel was associated with more beneficial clinical parameters and was identified as an independent prognostic factor. We conclude that the classical oligodendroglioma genotype provides additional prognostic data beyond clinical characteristics, morphology and tumor grade.

## Introduction

Recent advances in molecular techniques have provided important insights into the biology of gliomas, associating specific aberrations in molecular pathways with histological tumor entities. Such molecular biomarkers offer clinical useful information beyond diagnosis. Two recent trials showed improved outcome for patients with IDHmut-codel anaplastic oligodendrogliomas treated with radiotherapy and early adjuvant chemotherapy compared to non-codeleted tumors [1, 2]. Further, the effect of radiotherapy plus chemotherapy with procarbazine, CCNU and vincristine appeared to be largest for oligodendroglial tumors and IDHmut tumors [3]. In the recent EORTC trial of low-grade gliomas (LGG) comparing standard radiotherapy with primary temozolomide chemotherapy, a significant impact of radiotherapy on progression-free survival was found only in IDHmut LGG with intact 1p19q [4]. Thus, there is growing evidence that biomarkers are of prognostic and predictive importance in LGG and play a major role in rational decision making.

In the recent WHO classification of brain tumors, tumor diagnosis is based on both molecular and histological features [5]. The diagnosis of oligodendroglioma is restricted to IDHmut-codel tumors while tumors with oligodendroglioma morphology with unknown or incomplete genetic status are classified as oligodendroglioma NOS. Oligoastrocytomas have disappeared and are, with the aid of molecular markers, now classified as either astrocytomas or oligodendrogliomas. Of note, oligodendroglioma diagnosis based on morphology alone has also been subject to inter-observer variability, adding heterogeneity to this tumor type [6]. Thus, oligodendroglial tumors classified by the previous WHO classification consist of a heterogeneous group of oligodendrogliomas and astrocytomas [5].

Any new classification system brings up the question of the need of reclassification for patients still alive. As many centers have favored watchful waiting in patients with morphological oligodendroglioma, especially after extensive surgery, identifying patients with a more aggressive biology may be warranted. For these patients, and especially for patients with short or moderate follow-up times since the modified WHO 2016 classification, treatment strategies may depend upon a revised diagnosis. A pragmatic option would be to wait until progression or transformation occurs in these patients, and to perform molecular screening prior to new event-driven treatment decisions. However, this may be suboptimal since treatment does not appear to be as effective after progression as during early course of disease. In fact, the extensive later cross-over could not compensate for the large treatment effect in LGG of early treatment of radiotherapy followed by PCV [3]. Thus, identifying patients with the highest risk of progression seems warranted to optimize outcome by initiating treatment before malignant transformation occurs.

For identification of high-risk patients within the heterogeneous group of oligodendroglial tumors, it may be possible to use the established prognostic clinical variables for survival in LGG [7] together with IDHmut-codel. Our aim was to test this hypothesis in an unselected cohort of morphologically classified oligodendroglial tumors. We performed a retrospective review of oligodendroglial tumors operated at our hospital since 1983. Of a total of 214 oligodendroglial tumors, 64 tumors were IDHmut-codel and 150 had unknown or incomplete molecular profile. Here we present the prognostic impact of IDHmut-codel in this unselected cohort together with established prognostic clinical parameters for survival.

## Material and methods

### Patient population

We included patients 18 years or older operated at our hospital for an oligodendroglioma or oligoastrocyoma. The oldest cases operated between 1983–2001 were recruited from previously described low-grade glioma cohorts [8]. In addition, we collected clinical data for all patients operated between 2001–2015 with tumors classified as WHO grade II or III oligodendrogliomas or oligoastrocytomas according to the WHO 2007 classification [9]. The regional ethics committee, Regionala Etikprövningsnämnden Uppsala, approved the study protocol (Dnr 2005/1:5 and 2015/210). Data were collected retrospectively and anonymously and therefore no informed consent from patients included in this study was needed.

A total of 214 patients were enrolled. Tumor diagnosis was based on histological diagnosis or combined histological and molecular diagnosis. Consistent with the study aim, no additional molecular analysis or re-evaluation of histological diagnosis was performed for the purpose of this study.

### Molecular analyses

Mutated isocitrate dehydrogenase 1 (IDH1) R132H protein was detected using a monoclonal mouse antibody targeting the mutated IDH1 R132H protein (mIDH1R132) as described previously [10]. Immunostaining with mIDH1R132 has been part of the diagnostic procedure at our hospital since 2010, while IDH mutation analysis for tumors that are immunonegative for mIDH1R132 is not routinely performed. Detection of losses of chromosomal arms 1p and 19 was performed by fluorescent *in situ* hybridization analysis (FISH) for tumors operated between 1983–2001, as previously described [8]. In summary, the commercially purchased probes used for hybridization were Zytolight SPEC 1p36/1q25 and 19q13/19p13 dual color probes (Nordic BioSite, Sweden). Slides were assessed under a fluorescence microscope (Olympus BX 50 Deutschland GmbH) and a minimum of 100 non-overlapping nuclei was counted per hybridization [11]. A tumor was considered deleted if >50% of the nuclei harbored two signals of the reference probe, but only one signal of the target probe. Since 2013, a multiplex ligation-dependent probe amplification (MPLA) based assay (MRC-Holland) using Coffalayser.Net (2012) for analysis has been used for detection of losses of the chromosomal arms 1p and 19q at our hospital. Validation of the MPLA method was performed as previously described [12].

### Clinical data collection

A retrospective chart review was performed for all 214 patients with collection of the following data from the patient records and from CT or MRI scans of the brain: date and type of first symptoms, patient age at disease onset, date of operation, date of death, tumor location (specific lobe, 2 lobes or ≥ 3 lobes), preoperative performance status according to Karnofsky Performance Status (KPS) categorized as <90 or ≥ 90, date and type of surgery (biopsy, resection). Because radiological images were not available for older cases, the parameters tumor volume and extent of resection could not be assessed. Total survival was defined as the time point between first symptoms and date of death or end of follow-up (7 July 2016) and postoperative survival was defined as the time between surgery and date of death or end of follow-up. Follow-up time was from surgery to death or end of study. Data concerning time of death and cause of death were collected from central health authorities (the National Cause of Death Register Data).

### Statistical analysis

The statistical calculations were performed in Statistica, version13.2 (StatSoft, Inc. Tulsa, OK, USA) and in JMP version 13 (SAS software). Differences in distribution of clinical variables between the two groups were calculated by non-parametric tests: Mann–Whitney U test for continuous and categorical variables and Fisher exact two-tailed test for proportions. Survival was estimated by Kaplan-Meier method and Log-rank test. Multivariate survival analysis was performed by proportional hazard (Cox) regression. For selection of parameters in the multivariate model, we decided to include unrelated variables a priori, based on clinical experience and literature supporting the prognostic impact of these variables. Thus, the following parameters were used: age at onset (≥ 40 or < 40 years) [7, 13–15], gender [13], neurological deficit [7] or personality change [15], KPS score ≥ 90 or < 90 [14, 15], type of surgery [13, 14, 16, 17] (i.e. biopsy versus resection), WHO grade (II or III) [18] and molecular tumor marker status [19–21] (IDHmut-codel versus unknown/incomplete molecular profile). In addition, we tested the variable tumor location (frontal versus non-frontal) for which there is somewhat less convincing support as a prognostic factor in the literature [14]. Frontal (or other specific lobe) tumor location was defined as unilateral tumor growth restricted to the frontal lobe. Tumors involving more than one lobe were multilobar tumors and were categorized as 2 lobes or ≥3 lobes respectively. A p-value of <0.05 was considered statistical significant.

## Results

### Tumor diagnosis

Based on the availability of molecular markers, we divided tumors into IDHmut-codel oligodendrogliomas (grade II, n = 42; grade III, n = 22) or oligodendroglial tumors with unknown or incomplete molecular profile (NOS) (grade II, n = 82; grade III, n = 68). Table 1 shows the distribution of the molecular markers in these two groups.

**Table 1:** Distribution of molecular markers in the entire study sample of 214 oligodendroglial tumors.

Histological diagnosis	IDH-mut protein yes/no/unknown	1p19q codeletion yes/no/unknown
**Grade II & III**		
Oligo & Oligoastro (n = 214)	99/25/90	78/42/94
**Grade II (n = 124)**		
IDHmut-codel Oligo	42/0/0	42/0/0
Oligo & Oligoastro NOS	24/11/47	3/32/47
**Grade III (n = 90)**		
IDHmut-codel Oligo	22/0/0	22/0/0
Oligo & Oligoastro NOS	11/14/43	11/10/47

### Clinical features

#### Unselected patient population

Table 2 presents an overview of the clinical characteristics of patients with IDHmut-codel oligodendrogliomas and oligodendroglial tumors NOS. In S1 and S2 Tables the results for grade II and grade III tumors are presented separately. The study comprised 123 males (57%) and 91 females (43%). The mean age ±SD was 46.2 ±15.0 years, with younger age for patients with grade II tumors compared to grade III tumors (42.2 ±13.3 vs. 51.8 ±15.7 years; p<0.001). Patients with IDHmut-codel oligodendrogliomas were younger than patients with oligodendroglial tumors NOS. (Table 2). Epileptic seizures were the most common initial symptoms and occurred in 122 of all 214 patients (57%). Seizures were more common in patients with IDHmut-codel oligodendrogliomas, whereas neurological deficits and/or change of personality were more frequent in patients with oligodendroglial tumors NOS. (Table 2). Frontal lobe tumor location was most common for tumors restricted to one lobe and more often found in IDHmut-codel oligodendrogliomas than in oligodendroglial tumors NOS. (Table 2 and S1 Table).

**Table 2:** Distribution of clinical features for 1p19q codeleted oligodendrogliomas compared to oligodendroglial tumors with unknown or incomplete molecular profile.

	Grade II & III IDHmut-codel Oligo	Grade II & III Oligo & Oligoastro NOS	p-value
Number of patients, n	64	150	
Gender, n (%)			0.2
Male	41 (64.1)	82 (54.7)	
Female	23 (35.9)	68 (45.3)	
Mean age, years ±SD	41.9 ±12.0	48.0 ±15.8	0.01*
Age Category years, n (%)			0.009*
15–20	1 (1.6)	3 (2.0)	
21–30	12 (18.7)	20 (13.3)	
31–40	20 (31.2)	36 (24.0)	
41–50	16 (25.0)	27 (18.0)	
51–60	11 (17.2)	22 (14.7)	
61–70	3 (4.7)	30 (20.0)	
71–80	1 (1.6)	11 (7.3)	
81–90		1 (0.7)	
Seizures as first symptom, n (%)	46 (71.9)	76 (50.7)	0.04*
Neurological deficits or change of personality, n (%)	9 (14.1)	45 (30.0)	0.02*
KPS, n (%)			0.01*
<90	12 (18.8)	60 (40.0)	
≥ 90	52 (81.2)	90 (60.0)	
Tumor location, n (%)			0.1
Frontal	31 (48.4)	49 (32.6)	0.03*
Temporal	3 (4.7)	19 (12.7)	0.1
Parietal	5 (7.8)	9 (6.0)	0.8
Occipital	0	4 (2.7)	0.3
Insula	1 (1.6)	1 (0.7)	0.5
Corpus callosum	0	5 (3.3)	0.3
Central	0	3 (2.0)	0.6
Cerebellum	0	2 (1.3)	1.0
≥3 lobes	14 (21.9)	24 (16.0)	0.3
frontal-temporal-insular	11	18	
frontal-temporal-parietal	1	4	
temporal-parietal-occipital	1	2	
multiple lobes and central	1	0	
2 lobes	10 (15.6)	34 (22.6)	0.3
frontal-insular	4	6	
frontal-parietal	2	5	
frontal-temporal	0	2	
bifrontal	0	4	
temporal-insular	1	8	
temporal-occipital	1	8	
temporal-parietal	1	0	
parietal-occipital	1	1	
Surgery, n (%)			0.5
Resection	53 (82.8)	117(78.0)	
Biopsy	11 (17.2)	33 (22.0)	
Time first symptom-surgery, days median (IQR)	96 (54–222)	61 (27–182)	0.03*
Follow-up time, years median (IQR)	5.0 (2.6–9.6)	3.1 (1.5–5.7)	<0.01*

IQR = Inter Quartile Range,

* = significant

#### Selection of cases and individually matched controls

Due to the imbalance between the two groups regarding major prognostic factors, we validated our data in a selected sample consisting of cases and matched controls. For this procedure, all 64 patients with IDHmut-codel oligodendrogliomas were selected as cases while 64 individually matched controls for age and KPS were selected from the 150 patients with oligodendroglial tumors NOS. The selection of controls for each specific case was performed blindly by one of the authors (ASJ) who had no access to the survival data. The distribution of clinical features for these two groups matched by age and KPS are presented in S3 Table.

### Surgery

Forty-four patients (21%) underwent biopsy, 170 patients (79%) had tumor resection (Table 2). The time to surgery, defined as the time period between first symptoms and operation, was shorter for patients with grade III (median 56 days) than grade II tumors (median 128 days) (p<0.001). Also, the time to surgery was shorter in case of oligodendroglial tumors with unknown or incomplete molecular profile compared to IDHmut-codel oligodendrogliomas (Table 2).

### Survival analysis

**Fig 1:**
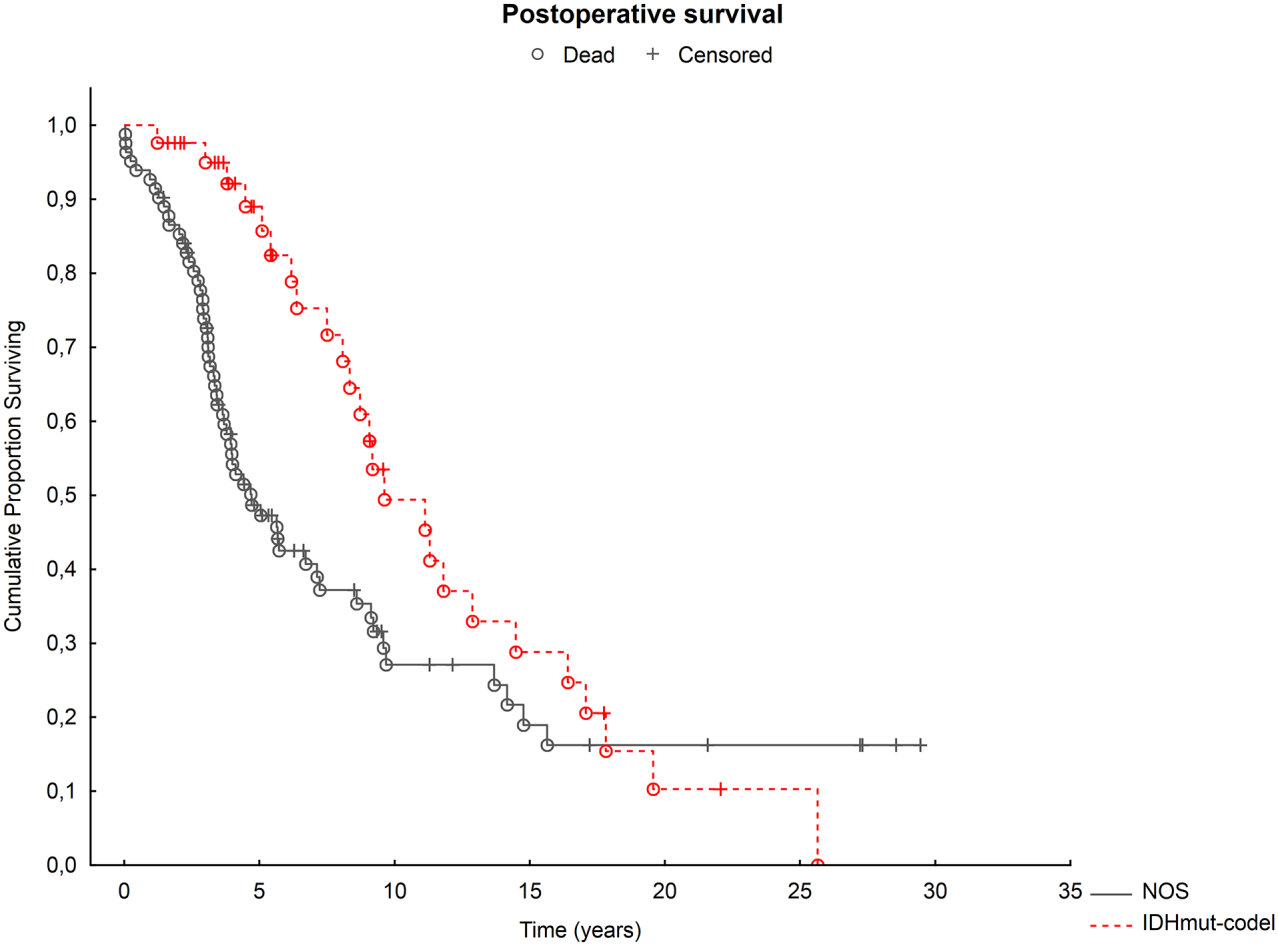
Postoperative survival in IDHmut-codel oligodendrogliomas compared to oligodendroglial tumors with unknown or incomplete molecular profile, NOS. Red circle: IDHmut-codel oligodendrogliomas. Black circle: oligodendroglial tumors with unknown or incomplete molecular profile, NOS. Censored (still alive) patients are indicated by + and dead patients by o.

The median total survival in the whole group (n = 214) was 4.2 years (Inter Quartile Range [IQR] 2.3–9.2), median postoperative survival was 3.6 years (IQR 2.0–7.2). For the combined group of grade II and III tumors, as well as for grade II and grade III separately, the median total and postoperative survival was longer in IDHmut-codel oligodendrogliomas compared to oligodendroglial tumors with unknown or incomplete molecular profile (Fig 1). Table 3 summarizes the median postoperative survival for the different groups. A similar pattern was found for total survival; median total survival in the group of IDHmut-codel oligodendrogliomas was 6 years (IQR 2.9–11.2) and 3.7 years (IQR 1.8–7.3) for patients with oligodendroglial tumors with unknown or incomplete molecular profile (p<0.001). Note, the 50% survival rate was not reached for the group of IDHmut-codel oligodendrogliomas and for the group of grade III IDHmut-codel oligodendrogliomas (Table 3). The favorable impact of IDHmut-codel on postoperative survival was confirmed analyzing cases with IDHmut-codel oligodendrogliomas in comparison with controls with oligodendroglial tumors NOS individually matched for age and KPS (results shown in S4 Table).

**Table 3:** Postoperative survival estimated by Kaplan-Meier method and Log-rank test in 214 oligodendroglial tumors.

Diagnosis	Censored n, (%)	Postoperative survival (years)		p-value
		Median	IQR	
Grade II & III (n = 214)	92 (43.0)			
IDHmut-codel Oligo (n = 64)	38 (59.4)**	5.0	2.6–9.6	<0.001*
Oligo NOS (n = 150)	54 (36.0)	3.1	1.5–5.7	
Grade II (n = 124)	43 (34.7)			
IDHmut-codel Oligo (n = 42)	17 (40.5)	6.9	3.8–11.3	0.03*
Oligo NOS (n = 82)	26 (31.7)	4.0	2.8–8.5	
Grade III (n = 90)	49 (54.4)			
IDHmut-codel Oligo (n = 22)	21 (95.5)**	2.6	2.1–4.9	<0.001*
Oligo NOS (n = 68)	28 (41.2)	2.1	0.9–4.6	

* significant;

** 50% survival rate not reached, IQR = Inter Quartile Range

### Predictors of survival

Regression analysis with the aim to identify predictors of survival was performed in the whole group of 214 patients (92 patients = 43% censored) using the variables previously described (Table 4). We identified the following independent predictors for postoperative survival: age at onset (age ≥ 40 years: HR 1.78, p = 0.004), absence of neurological deficit or personality change (HR 0.57, p = 0.01), frontal tumor location (HR 0.64, p = 0.03) and molecular status of the tumor (IDHmut-codel: HR 0.50, p = 0.002). Similar results with identification of identical prognostic factors were obtained for total survival. Tumor malignancy grade did not significantly predict survival in the model (Table 4). However, it should be noted that the inclusion of grade III tumors was not evenly during the entire study period and that these tumors were mainly included after 2005. Therefore, a second regression analysis was performed in which we analyzed survival for patients included between 2005 and 2016 (n = 135). The results from this adapted model were similar, showing no significant impact of tumor grade on survival.

**Table 4:** Cox regression model for postoperative survival in the entire cohort of 214 oligodendroglial tumors.

	Multivariate Cox regression			
	N/n	Hazard Ratio	0.95 CI	p-value
Age ≥ 40 vs. < 40 years	122/92	1.78	1.20–2.63	0.004*
KPS ≥ 90 vs. < 90	142/72	0.89	0.58–1.35	0.6
Gender: male vs. female	123/91	1.21	0.83–1.77	0.3
WHO grade: II vs. III	124/90	0.84	0.56–1.30	0.4
Surgery: Biopsy vs. resection	44/170	0.87	0.57–1.35	0.5
Frontal tumor location	80/134	0.64	0.43–0.96	0.03*
Absence of neurological deficit or personality change	160/54	0.57	0.36–0.90	0.01*
Molecular tumor status: IDHmut-codel vs. NOS	64/150	0.50	0.31–0.78	0.002*

N/n = number of patients in each category,

*significant, CI = Confidence interval.

For ten patients, the tumor was 1p19q codeleted but without IDH1 mutation detected by the mIDH1R132 antibody. Since the association between 1p19q codeletion and IDH mutation is very strong [22], we performed another post-hoc analysis, with these ten samples included in the IDHmut-codel group in the Kaplan Meier and Cox regression. These corrections did not alter the results as described for the original cohort of 64 IDHmut-codel oligodendrogliomas and 150 oligodendroglial tumors with unknown or incomplete molecular profile.

## Discussion

In this study, we found that the presence of IDHmut-codel is an independent predictor of survival in patients with tumors of oligodendroglial morphology. Furthermore, this molecularly defined subclass seems to be a more important predictor of survival than WHO grade in these tumors. We could also confirm an association between IDHmut-codel tumors and specific clinical parameters. Our results show that IDHmut-codel tumors are overrepresented in patients with frontal tumor location, younger age, without neurological deficit or personality change and with seizure at presentation. These differences in clinical characteristics may be used to identify high-risk patients amongst patients with oligodendroglial tumors that have not been molecularly characterized.

From a clinical perspective our findings indicating that IDHmut-codel is a strong prognostic predictor, argue for introducing clinical routines for retrospective molecular screening of all tumors with oligodendroglial morphology, irrespective of the course of disease for these patients. In fact, our findings stress the importance of the molecular pathway rather than the malignancy grade for outcome in patients with lower-grade (WHO grade II and III) gliomas [23]. The validity of our results is strengthened by what is known from the literature regarding tumor location, i.e. the strong predilection of oligodendrogliomas harboring 1p19q codeletion for the anterior part of the brain [24–26]. In addition, our data are in line with previous studies showing that seizures as initial symptom have a positive impact on survival [27–31] whereas the presence of neurological deficits [7, 32] and cognitive change [15] are unfavorable prognostic factors in this patient group. Also, the unfavorable impact of high age on survival is well described [7, 13–15, 33–35]. Taken together, our study demonstrates the importance of a number of well-established clinical prognostic factors for survival in a heterogeneous group of oligodendroglial tumors.

By combining histological features with molecular markers, the new WHO classification of brain tumors has made an important step forward towards personalized glioma treatment [5]. In clinical practice, however, there are still a number of challenges that need to be addressed before wide-scale routine molecular testing for gliomas will become reality at many centers. Very few studies have addressed the dilemma that molecular tests may not be routinely available or, if available, are not always used according to current recommendations. A recent Australian survey of attitudes amongst neuro-oncology clinicians found that only 25% of the respondents used routinely tests for 1p19q status in diffuse low-grade gliomas at their institution, although 69% were of the opinion that all patients should be tested [36]. The consequences of having an incomplete tumor diagnosis lie in a falsely favorable assessment of the expected natural course of disease with direct consequences for treatment strategy. For patients with oligodendroglial tumors with long, but also extremely varying life expectancy, these risks are not negligible. It is reasonable to believe that many patients worldwide are followed-up up at local hospitals where the indication for re-classification is not systematically considered.

There are a number of limitations with the present study that need to be considered. Although we made an effort to identify all eligible patients operated at our hospital and to include all relevant clinical information, data collection was performed retrospectively and selection bias cannot be excluded. For example, for the oldest patients CT or MRI scans were not available, and the evaluation of tumor location in these cases was based on radiological reports only. As described in the Results Section, we encountered an uneven inclusion of anaplastic tumors over time. To overcome the imbalance between the two groups for major prognostic factors, we performed an additional case-control analysis comparing all 64 IDHmut-codel cases with 64 oligodendrogial tumors NOS individually matched for age and KPS. The favorable impact of IDHmut-codel on postoperative survival in these tumors was confirmed in this analysis. It should be noted, however, that other non-identified factors may influence patient outcome that have not been analyzed in our model. Finally, another limitation of our study is that mutated IDH1R132H protein was detected using immunohistochemistry only. Since the vast majority of IDH mutations involve codon 132 of the IDH1, this method will detect around 90% of IDH mutations and therefore the remaining 10% (mainly IDH2 mutations) will be missed [21]. Taking into account the strong association between 1p19q codeletion and IDH mutation [22], some underestimation of IDH mutations in this series is to be expected. This is particularly likely for the ten tumors with 1p19q codeletion but *without* IDH1 mutated protein that was included in the group of tumors unknown/incomplete molecular profile. The post-hoc sensitivity analysis, including these patients in the IDHmut-codel group, did however not alter the results.

In conclusion, this study demonstrates that the classical oligodendroglioma genotype of IDHmut-codel provides additional prognostic information beyond tumor grade, morphology and clinical characteristics. Our results speak in favor for retrospective molecular screening of all tumors with oligodendroglial morphology, irrespective of the course of disease for these patients. In centers where routine molecular profiling is not available, the selection of high-risk patients based on clinical characteristics is possible, albeit unspecific and crude. As such patient with older age, non-frontal tumor location and presenting with neurological deficits and/or personality change instead of epileptic seizures, are at risk for having non-oligodendroglioma genotype with worse prognosis.

## Supporting information

S1 Table Distribution of clinical features for WHO grade II IDH-mutated 1p19q codeleted oligodendrogliomas compared to WHO grade II oligodendroglial tumors with unknown or incomplete molecular profile. IQR = Inter Quartile Range, * = significant.
(DOCX)

S2 Table Distribution of clinical features for WHO grade III IDH-mutated 1p19q codeleted oligodendrogliomas compared to WHO grade III oligodendroglial tumors with unknown or incomplete molecular profile. IQR = Inter Quartile Range, * = significant.
(DOCX)

S3 Table Distribution of clinical features in the two groups, IDHmut-codeleted and oligodendroglial tumors with unknown or incomplete molecular profile NOS, matched for age and KPS. (DOCX)

S4 Table Postoperative survival estimated by Kaplan-Meier method and Log-rank test in the two groups, matched for age and KPS. ** 50% survival rate not reached, IQR = Inter Quartile Range.
(DOCX)

S1 File The anonymized data set of the whole patient population in this study. (XLSX)

S2 File The anonymized data used in the regression analysis. (XLSX)

S3 File The anonymized data of the matched group. (XLSX)
