# Supplementary material for: Prognostic markers for survival in patients with oligodendroglial tumors; a single-institution review of 214 cases
Source: PLoS One. 2017 Nov 29;12(11):e0188419. doi: 10.1371/journal.pone.0188419 (PMC5706698; doi:10.1371/journal.pone.0188419)
Supplement: S1 Table — IQR = Inter Quartile Range, * = significant. (DOCX) [file pone.0188419.s001.docx]

**S1 Table.** Distribution of clinical features for WHO grade II IDH-mutated 1p19q codeleted oligodendrogliomas compared to WHO grade II oligodendroglial tumors with unknown or incomplete molecular profile.

|  | Grade II IDHmut-codel Oligo | Grade II Oligo & Oligoastro NOS | p-value |
| --- | --- | --- | --- |
| Number of patients, n | 42 | 82 |  |
| **Gender,** n (%) |  |  | 0.6 |
| Male | 28 (66,7) | 49 (59.8) |  |
| Female | 14 (33.3) | 33 (40.2) |  |
| Mean age, years ±SD | 40.4 ±11.5 | 43.2 ±14.1 | 0.4 |
| Seizures as first symptom, n (%) | 34 (81.0) | 56 (68.3) | 0.2 |
| Neurological deficits or change of personality, n (%) | 3 (7.1) | 15 (18.3)) | 0.1 |
| **KPS, n (%)** |  |  | 0.2 |
| <90 | 9 (21.4) | 30 (36.6) |  |
| ≥90 | 33 (78.6) | 52 (63.4) |  |
| **Tumor location (n) (%)** |  |  | 0.09 |
| Frontal | 22 (52.3) | 26 (31.7) | 0.03* |
| Temporal | 0 | 8 (9.8) | 0.05 |
| Parietal | 4 (9.5) | 3 (3.7) | 0.2 |
| Occipital | 0 | 2 (2.4) | 0.5 |
| Corpus callosum | 0 | 4 (4.9) | 0.3 |
| Central | 0 | 3 (3.7) | 0.6 |
| ≥3 lobes | 10 (23.8) | 18 (22.0) | 0.8 |
| 2 lobes | 6 (14.3) | 18 (22.0) | 0.3 |
| **Surgery,** n (%) |  |  | 0.7 |
| Resection | 33 (78.6) | 61 (74.4) |  |
| Biopsy | 9 (21.4) | 21 (25.6) |  |
| Time first symptom-surgery, days median (IQR) | 156 (65-510) | 91 (30-393) | 0.07 |
| Follow-up time, years median (IQR) | 6.9 (3.8-11.3) | 5.1 (3.1-9.5) | 0.008* |

IQR = Inter Quartile Range, * = significant
