# Supplementary material for: Prognostic markers for survival in patients with oligodendroglial tumors; a single-institution review of 214 cases
Source: PLoS One. 2017 Nov 29;12(11):e0188419. doi: 10.1371/journal.pone.0188419 (PMC5706698; doi:10.1371/journal.pone.0188419)
Supplement: S2 Table — IQR = Inter Quartile Range, * = significant. (DOCX) [file pone.0188419.s002.docx]

**S2 Table.** Distribution of clinical features for WHO grade III IDH-mutated 1p19q codeleted oligodendrogliomas compared to WHO grade III oligodendroglial tumors with unknown or incomplete molecular profile.

|  | Grade III IDHmut-codel Oligo | Grade III Oligo & Oligoastro NOS | p-value |
| --- | --- | --- | --- |
| Number of patients (n) | 22 | 68 |  |
| **Gender,** n (%) |  |  | 0.5 |
| Male | 13 (59.1) | 33 (48.5) |  |
| Female | 9 (40:9) | 35 (51.5) |  |
| Mean age ±SD (years) | 44.9 ±12.7 | 53.8 ±16 | 0.01* |
| Seizures as first symptom, n (%) | 12 (54.5) | 20 (29.4) | 0.04* |
| Neurological deficits or change of personality, n (%) | 6 (27.0) | 30 (44.1) | 0.2 |
| **KPS** **n (%)** |  |  | 0.03* |
| <90 | 3 (13.6) | 30 (44.1) |  |
| ≥90 | 19 (86.4) | 38 (55.9) |  |
| **Tumor location, n (%)** |  |  | 0.7 |
| Frontal | 9 (40.9) | 23 (33.8) | 0.6 |
| Temporal | 3 (13.6) | 11 (16.2) | 1.0 |
| Parietal | 1 (4.5) | 6 (8.8) | 0.7 |
| Occipital | 0 | 2 (2.9) | 1.0 |
| Insula | 1 (4.5) | 1 (1.5) | 0.4 |
| Corpus callosum | 0 | 1 (1.5) | 1.0 |
| Cerebellum | 0 | 2 (2.9) | 1.0 |
| ≥3 lobes | 4 (18.2) | 6 (8.8) | 0.3 |
| 2 lobes | 4 (18.2) | 16 (23.5) | 0.8 |
| **Surgery,** n (%) |  |  | 0.5 |
| Resection | 20 (90.9) | 56 (82.4) |  |
| Biopsy | 2 (9.1) | 12 (17.6) |  |
| Time first symptom-surgery, days median (IQR) | 66 (33-92) | 48 (24-106) | 0.6 |
| Follow-up time, years median (IQR) | 2.6 (2.1-4.9) | 2.1 (0.9-4.6) | 0.05 |

IQR = Inter Quartile Range, * = significant
