# Supplementary material for: Prognostic markers for survival in patients with oligodendroglial tumors; a single-institution review of 214 cases
Source: PLoS One. 2017 Nov 29;12(11):e0188419. doi: 10.1371/journal.pone.0188419 (PMC5706698; doi:10.1371/journal.pone.0188419)
Supplement: S3 Table — (DOCX) [file pone.0188419.s003.docx]

**S3 Table.** Distribution of clinical features in the two groups, IDHmut-codeleted and NOS, matched for age and KPS.

|  | Grade II & III IDHmut-codel Oligo | Grade II & III Oligo & Oligoastro NOS | p-value |
| --- | --- | --- | --- |
| Number of patients, n | 64 | 64 |  |
| Gender, n (%) |  |  | 0.7 |
| Male | 41 (64) | 38 (59) |  |
| Female | 23 (36) | 26 (41) |  |
| Mean age, years ±SD | 41.9 ±12 | 41,8 ±12 | 0.9 |
| Seizures as first symptom, n (%) | 46 (72) | 39 (61) | 0.3 |
| Neurological deficits or change of personality, n (%) | 9 (14) | 11 (17) | 0.8 |
| KPS |  |  | 0.7 |
| <90 | 12 | 12 | 1.0 |
| ≥ 90 | 52 | 52 |  |
| Frontal tumor location, n (%) |  |  |  |
| WHO grad, n (%) |  |  | 0.7 |
| II | 42 | 39 |  |
| III | 22 | 25 |  |
| Follow-up time, median (25th-75th percentile) | 5.0 (2.6-9.6) | 4.2 (2.8-6.9) | 0.05 |
