# Supplementary material for: Prognostic markers for survival in patients with oligodendroglial tumors; a single-institution review of 214 cases
Source: PLoS One. 2017 Nov 29;12(11):e0188419. doi: 10.1371/journal.pone.0188419 (PMC5706698; doi:10.1371/journal.pone.0188419)
Supplement: S4 Table — ** 50% survival rate not reached, IQR = Inter Quartile Range. (DOCX) [file pone.0188419.s004.docx]

**S4 Table.** Postoperative survival estimated by Kaplan-Meier method and Log-rank test in the groups matched for age and KPS.

| Diagnosis | Censored n, (%) | Postoperative survival (years) | | p-value |
| --- | --- | --- | --- | --- |
|  |  | Median | IQR |  |
| IDHmut-codel Oligo (n=64) | 38 (59.4)** | 5.0 | 2.6 - 9.6 | 0.05 |
| Oligo NOS (n=64) | 32 (50.0) | 4.2 | 2.8 - 6.9 |  |

** 50% survival rate not reached, IQR = Inter Quartile Range
